# Corrigendum to “A New Neurocognitive Interpretation of Shoulder Position Sense during Reaching: Unexpected Competence in the Measurement of Extracorporeal Space”

**DOI:** 10.1155/2018/9104751

**Published:** 2018-01-09

**Authors:** Teresa Paolucci, Federico Zangrando, Giulia Piccinini, Federico Sciarra, Rocco Pallotta, Alice Mannocci, Giuseppe La Torre, Fabiano Bini, Franco Marinozzi, Stefano Gumina, Luca Padua, Vincenzo Maria Saraceni

**Affiliations:** ^1^Complex Unit of Physical Medicine and Rehabilitation, Sapienza University of Rome, Azienda Policlinico Umberto I, Piazzale Aldo Moro 5, 00185 Rome, Italy; ^2^Department of Public Health and Infectious Diseases, Sapienza University of Rome, Azienda Policlinico Umberto I, Piazzale Aldo Moro 5, 00185 Rome, Italy; ^3^Department of Mechanical and Aerospace Engineering, Sapienza University of Rome, Azienda Policlinico Umberto I, Piazzale Aldo Moro 5, 00185 Rome, Italy; ^4^Department of Orthopedics and Traumatology, Sapienza University of Rome, Azienda Policlinico Umberto I, Piazzale Aldo Moro 5, 00185 Rome, Italy; ^5^Department of Geriatrics, Neurosciences and Orthopedics, Division of Neurophysiopathology, Teaching Hospital “Agostino Gemelli”, Rome, Italy

In the article titled “A New Neurocognitive Interpretation of Shoulder Position Sense during Reaching: Unexpected Competence in the Measurement of Extracorporeal Space” [1], there were errors in the following sections.

In the Abstract, the sentence “*Results*. The shoulder had proprioceptive features that allowed it to memorize a reaching position and reproduce it (error of 1.22 cm to 1.55 cm in healthy subjects)” should be changed to “*Results*. The shoulder had proprioceptive features that allowed it to memorize a reaching position and reproduce it (error of 0.62 cm ± 0.57 cm in healthy subjects).”

In the Materials and Methods under the “Assessment of Error” subsection, the definition of “spatial error” reported in the Abstract and the correspondence of “spatial error” with “gap in precision” should be added. Therefore, the text reading “Both tests entailed six assessments: three reaching forward and three reaching back. At the end of each evaluation, the examiner noted the error by the subject, defined as the GAP in precision (cm)” should be changed to “Both tests entailed six assessments: three reaching forward and three reaching back. At the end of each evaluation, the examiner noted the error by the subject, defined as the spatial error or GAP in precision (cm).”

In Table 2, the note “b” refers the *p* value of Student's *t*-test for independent sample with equal variances not assumed. A note “g” is added for *p* value Student's *t*-test for paired samples. The corrected table is shown in this paper.

In the Results section, under the “Analysis of the Tests” subsection, the text reading “Between independent Tests 1 and 2 (*N* = 150, reaching total), both groups measured better blindfolded and in the active phase, with average errors of 0.47 ± 0.34 in the HG and 0.75 ± 0.65 in the IG in Test 1 and 0.38 ± 0.29 and 0.66 ± 0.45, respectively, for active modality and 0.50 ± 0.66 and 0.79 ± 0.68 for passive modality in Test 2” should be corrected to “Between independent Tests 1 and 2 (*N* = 150, reaching total), both groups measured better blindfolded and in the active phase, with average errors of 0.47 ± 0.34 in the HG and 0.75 ± 0.65 in the IG in Test 1 and 0.38 ± 0.29 and 0.66 ± 0.45, for passive modality, and 0.50 ± 0.66 and 0.79 ± 0.68 for active modality in Test 2.”

In the Discussion, the following two sentences should be changed.

(1) The text reading “Our results indicate that HG and IG subjects estimate an average error concerning the reaching movement that is required of 1.11 ± 1.16 in the HG and 1.82 ± 1.58 in IG (*p* < 0.001), suggesting that the shoulder has its own proprioceptive ability that is reduced in impingement syndrome” should be corrected to “Our results indicate that HG and IG subjects estimate an RGAP concerning the reaching movement that is required of 0.62 ± 0.57 in the HG and 1.01 ± 0.70 in IG (*p* < 0.001), suggesting that the shoulder has its own proprioceptive ability that is reduced in impingement syndrome.”

(2) The text reading “With regard to how the proprioceptive sense of the shoulder integrates visual control, our results indicate that subjects perform better in the blindfolded test as in HG as in IG; in particular, the average error was minor in the blindfolded test for reaching forward in Test 2 in the passive modality in both groups” should be corrected to “With regard to how the proprioceptive sense of the shoulder integrates visual control, our results indicate that subjects perform better in the blindfolded passive test as in HG as in IG; in particular, the average error was minor in the blindfolded test for reaching forward in Test 2 in the passive modality in both groups.”

## Figures and Tables

**Table 1:** Description of RGAP (mean ± SD and median with min–max) in the two groups stratifying by tests (Test 1 and Test 2 in active and passive modality) and overall (all tests).

Tests and modality	RGAP^a^ HG						RGAP^a^ IG						Test to compare	
													HG versus IG	
	*N*	Mean^d^	SD^d^	Median^d^	min–max^d^	*p*	*N*	Mean^d^	SD^d^	Median^d^	min–max^d^	*p*	*p* ^f^	Test
All tests														
Reaching-forward + reaching-back	450	0.62	0.57	0.5	0–3.39	-	450	1.01	0.7	0.92	0–3.44	-	**<0.001**	b
Reaching-forward	225	0.29	0.22	0.28	0.02–1.30	**<0.001** ^b^	225	0.46	0.3	0.49	0–1.62	**<0.001** ^g^	**<0.001**	b
Reaching-back	225	0.62	0.57	0.5	0–3.39		225	1.01	0.7	0.61	0–3.44		**<0.001**	b

Reaching-forward + reaching-back														
Test 1	150	0.47	0.34	0.28	0–1.33	0.352^e^	150	0.75	0.65	0.48	0–3.44	0.505^e^	**0.01**	b
Test 2 passive	150	0.38	0.29	0.23	0–1.22		150	0.66	0.45	0.33	0.11–1.83		**<0.001**	b
Test 2 active	150	0.5	0.66	0.19	0.03–3.39		150	0.79	0.68	0.35	0–2.78		**0.033**	c

Reaching-forward														
Test 1	75	0.4	0.32	0.28	0.02–1.3	**<0.001** ^e^	75	0.59	0.38	0.47	0.03–1.62	**0.016** ^e^	0.055	c
Test 2 passive	75	0.27	0.13	0.23	0–1.33		75	0.4	0.24	0.33	0.16–0.95		**0.022**	b
Test 2 active	75	0.19	0.1	0.19	0–0.39		75	0.37	0.23	0.35	0–0.76		**0.001**	b

Reaching-back														
Test 1	75	0.55	0.35	0.5	0.0–1.3	0.095^e^	75	0.91	0.82	0.61	0–3.44	0.224^e^	0.055	b
Test 2 passive	75	0.49	0.36	0.44	0–1.22		75	0.91	0.47	0.88	0.11–1.83		**0.001**	c
Test 2 active	75	0.8	0.82	0.56	0.06–3.39		75	1.2	0.73	1.17	0.06–2.78		0.075	c

^a^The RGAP reported the relative error computed considering the difference between the measure requested and the measure done by the patients on measure requested; ^b^Student's  *t*-test for independent sample with equal variances not assumed; ^c^Student's  *t*-test for independent sample with equal variances assumed; ^d^the mean and the median GAP are computed relativizing the absolute value (= the absolute number value) of the gap value of the measure requested; ^e^*p* value of MANOVA test; ^f^*p* value for two independent samples; the specific test is reported in the last column; ^g^*p* value for Student's *t*-test for paired samples; *N*: number of measurements tested.
